# The pathogenesis of COPD and IPF: Distinct horns of the same devil?

**DOI:** 10.1186/1465-9921-13-3

**Published:** 2012-01-11

**Authors:** Marco Chilosi, Venerino Poletti, Andrea Rossi

**Affiliations:** 1 Department of Pathology, University of Verona, Italy; 2 Department of Diseases of the Thorax, Morgagni Hospital, Forlì, Italy; 3 Pulmonary Division, Verona General Hospital, Italy

**Keywords:** COPD, IPF, precursor cell senescence, telomere dysfunction, Wnt, Notch, Caveolin-1

## Abstract

New paradigms have been recently proposed in the pathogenesis of both chronic obstructive pulmonary disease (COPD) and idiopathic pulmonary fibrosis (IPF), evidencing surprising similarities between these deadly diseases, despite their obvious clinical, radiological and pathologic differences. There is growing evidence supporting a "double hit" pathogenic model where in both COPD and IPF the cumulative action of an accelerated senescence of pulmonary parenchyma (determined by either telomere dysfunction and/or a variety of genetic predisposing factors), and the noxious activity of cigarette smoke-induced oxidative damage are able to severely compromise the regenerative potential of two pulmonary precursor cell compartments (alveolar epithelial precursors in IPF, mesenchymal precursor cells in COPD/emphysema). The consequent divergent derangement of signalling pathways involved in lung tissue renewal (mainly Wnt and Notch), can eventually lead to the distinct abnormal tissue remodelling and functional impairment that characterise the alveolar parenchyma in these diseases (irreversible fibrosis and bronchiolar honeycombing in IPF, emphysema and airway chronic inflammation in COPD).

## Introduction

Chronic obstructive pulmonary disease (COPD) and idiopathic pulmonary fibrosis (IPF) are two severe multifactorial pulmonary disorders characterised by quite distinct clinical and pathological features. COPD is characterised by a poorly reversible and progressive airflow limitation that is determined by the concurrence of airways inflammation and emphysema (from now on both included in the acronym COPD)[1,2], whereas in IPF a restrictive pattern of lung volume abnormality is associated with impaired diffusion capacity [3]. At imaging and pathological examinations COPD and IPF exhibit different appearances, as far as the involved pulmonary regions (upper lobes versus lower lobes), and the occurring parenchymal modifications are concerned (alveolar emphysematous dilation and bronchiolar inflammation in COPD, versus interstitial fibrosis and honeycombing in IPF)[1-3]. Finally, the incidence and prevalence of the two diseases are quite different, since IPF is considered a rare condition (although incidence and prevalence are both rising due to improved diagnostic tools), whereas the COPD prevalence is very high, although variable in different risk populations [4,5].

Nevertheless, a number of similarities can be recognised between the two disorders. Firstly, both COPD and IPF are chronic and progressive diseases of elderly people (with male predominance), that severely affect the lung function, and both are related to long term inhalation of external noxious agents (mainly tobacco smoking)[3,4,6,7]. Secondly, in both diseases a progressive loss of alveolar parenchyma takes place leading to severe impairment of respiratory function. Variants of pulmonary fibrosis associated with emphysema have been described, and these cases have been grouped in a newly defined syndrome of combined pulmonary fibrosis and emphysema (CPFE)[8]. In CPFE, lung volumes are commonly within normal limits due to the opposing effects of hyperinflation and fibrosis. The CPFE syndrome is more frequent in male smokers, and pulmonary hypertension can complicate all these disorders [8,9]. Finally, both IPF and COPD are associated with an increased risk of cancer development, and several lines of evidence suggest that this increase is independent from the effect of cigarette smoking [10,11].

Despite the great deal of research, effective treatments are lacking for both COPD and IPF. This can be a consequence, at least in part, of the limited understanding of their pathogenesis, despite the overwhelming plethora of studies and theories proposed so far. Interestingly, for both diseases a gradual shift from "inflammatory-based" pathogenic theories to more complex approaches occurred in recent years [12,13]. In this evolving scenario, a variety of concurrent underlying pathogenic mechanisms have been proposed for these diseases, including oxidative stress, protease/anti-protease imbalance, abnormal healing after damage, deranged remodelling, enhanced apoptosis, and others [14-18].

### Accelerated senescence in the pathogenesis of IPF and COPD

The most striking new information linking the pathogenesis of IPF and COPD relates to their proposed inclusion within the category of diseases with alveolar senescence and lung "premature aging" [19-33]. The senescence hypothesis for both COPD and IPF pathogenesis is supported by a variety of studies demonstrating telomere length abnormalities, as well as the in situ expression of senescence-related cell-cycle regulators (p21^WAF1 ^and p16^INK4a ^)[19,25,34,35]. The role of cell senescence is particularly evident in familial IPF, where nearly 10% of cases harbour mutations of one of the two key components involved in telomere lengthening: the reverse transcriptase component TERT and the RNA template component TERC [29,30]. In addition, about 20% of patients suffering for dyskeratosis congenita, a well characterized genetic disease caused by telomerase mutations, develop pulmonary fibrosis [36].

Although abnormalities directly affecting telomerase genes have not been demonstrated in COPD, telomere decreased length has been demonstrated in either lung cells or peripheral leukocytes in COPD patients, compared with control subjects [21,24-27]. The excess of telomere attrition further supports the concept of COPD as a systemic disorder of premature aging, as also suggested by the occurrence of relevant comorbidities, such as weight loss, osteoporosis, cardiovascular diseases, and depression, where premature senescence and telomere length abnormalities have been also documented [37-39]. Further experimental evidence has been recently provided that telomere length is a susceptibility factor in emphysema [40].

### Epithelial progenitor cell dysfunction in IPF

The pathogenic role of genetic abnormalities in IPF is much more evident than in COPD, and familial IPF has been recently included within the category of genetic diseases with "telomere dysfunction" together with acquired aplastic anaemia and dyskeratosis congenita [41-44]. These diseases are characterised by clinical and pathologic heterogeneity despite the similarity of underlying genetic defects affecting the telomere elongating mechanisms (mainly TERT or TERC mutations). In these diseases specific phenotypes are likely related to the progenitor cell type(s) involved as "weak spots" in disease development (e.g. haemopoietic precursors in aplastic anaemia)[45]. A correct telomere maintaining program in precursor cells is crucial to avoid degenerative disorders and anticipated aging, and short telomeres cause precursor cell failure in experimental systems [46,47]. In addition, it has been clearly established that the development of diseases with "telomere dysfunction" needs the contribution of both a genetic predisposing abnormality as well as an environmental factor in order to develop the entire disease phenotype [45]. Thus, the exposure to benzene or other toxic substances, is key for the development of aplastic anaemia, as it can be considered tobacco smoke (or exposure to other toxic substances) in IPF. A significant role might be also exerted by gender [48], as it could be also expected in cases of pulmonary fibrosis complicating X-linked dyskeratosis congenita [49]. Accordingly, most described cases of pulmonary fibrosis complicating dyskeratosis congenita are former-smoker and male [36,49,50].

Familial IPF cases have been linked not only to mutations of telomerase genes, but also to mutations in surfactant proteins coding genes (surfactant protein-C and A2)[51-54]. Chronic epithelial injury in these cases is likely related to protein abnormalities that can either impair the crucial functions of the surfactant, or induce endoplasmic reticulum stress and apoptosis [55]. Type-II pneumocytes, that are the main producers of surfactant proteins and are the epithelial precursors of alveolar parenchyma, are the specific target of cellular death produced by surfactant protein abnormalities.

**Figure 1:**
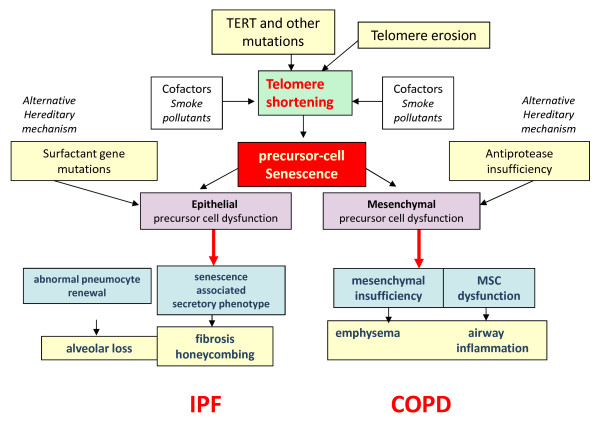
**Pathogenic scheme of IPF and COPD**. Summation of genetic and environmental factors underlay the abnormal renewal of either epithelial (left) or mesenchymal (right) alveolar components leading to parenchymal fibrotic obliteration and remodeling in IPF, or emphysematous changes and airway inflammation in COPD. The genetic background can be either hereditary (fully consistent with a "telomere dysfunction" as observed in familial IPF), or can variably provide a genetic susceptibility.

Thus different mechanisms can be responsible of the progressive loss of pneumocyte precursors in IPF, including accelerated senescence, surfactant abnormalities and endoplasmic reticulum stress, all potentially causing precursor cell exhaustion and abnormal alveolar re-epithelialisation [56-59]. The concurrent action of environmental factors such as the exposure to toxic substances, and especially tobacco smoking and/or pollution appear as necessary for developing the disease in both familial and sporadic IPF, although IPF can occur also in non smokers [6,60](Figure 1).

### Mesenchymal precursor cell insufficiency in COPD/emphysema

In COPD a large amount of data suggest that parenchymal remodelling and progressive dilation of alveolar spaces is related to decreased and/or deregulated production of extracellular matrix proteins, in particular elastin, with the eventual impaired capability to sustain connective and epithelial tissue repair. Connective tissue insufficiency can be either caused by genetic defects as observed in alpha-1 anti-trypsin deficiency (a well established genetically determined form of COPD/emphysema where elastin decrease can be directly related to the observed alveolar dilation, loss of elastic recoil and airflow obstruction), or it may be ascribed to ill defined causes inducing mesenchymal precursor cell senescence and progressive decrease of matrix protein production, as suggested in this review. Accordingly, senescence related markers in COPD are mainly demonstrable in mesenchymal cells (fibroblasts and endothelial cells) [61], and a variety of abnormalities have been described affecting pulmonary mesenchymal cells, including fibroblasts and endothelial cells in both human and experimental COPD [62-68](Figure 1).

### IPF and COPD: two distinct horns of the same devil?

All this taken into account, it is possible to hypothesise that in the pathogenesis of both COPD and IPF the main driving abnormality is the precocious senescence of pulmonary parenchyma.

But how can be reconciled this proposed similarity between the basic pathogenic mechanisms underlying COPD and IPF with the obvious diversity of their clinical and pathological presentations? A possible explanation can be searched in the diversity of the genetic alterations, including genetic/epigenetic inheritance or predisposing gene polymorphisms, compromising the renewal capacity of different target cells [69-71]. As proposed above, if in IPF the major target is likely the alveolar epithelial precursor cell (type II pneumocyte), in COPD the Achille's heel may be represented by mesenchymal precursor cells within the alveolar parenchyma.

Human and experimental studies provide strong evidence that molecular networks regulating parenchymal lung tissue renewal are perturbed in both COPD and IPF, although in different ways. Among these networks, particular relevance has been focused on the interplay between Notch and Wnt, two signalling pathways playing critical roles in epithelial and mesenchymal precursor cell maintenance and differentiation [72-78].

### Wnt and Notch pathway perturbation in IPF

In IPF the senescent phenotype seems to mainly affect the epithelial precursors of alveolar tissue (type-II pneumocytes), thus preventing a correct epithelial renewal at anatomical sites where mechanical stress and alveolar damage are expected to be maximal (lower/peripheral lung zones) [56,79]. Pneumocyte loss is followed, in this pathogenic scheme of IPF, by attempted tissue regeneration and exaggerated release of molecular signals (Wnt and Notch) triggering fibroblast proliferation and migration. Several human and experimental studies have confirmed that the Wnt-pathway is abnormally activated in IPF, and this notion is included in recent pathogenic models for IPF [17,56,80-82]. The relevance of abnormal Wnt-signalling activation in IPF is confirmed by the up-modulation of various Wnt-pathway molecular targets observed in IPF (MMP7, cyclin-D1 and others) [80], as well as by the demonstration that experimental fibrosis can be attenuated by the Wnt/β-catenin pathway blockade [83]. Accordingly, perturbation of the Wnt-pathway is directly related to abnormal myofibroblast activation and epithelial-mesenchymal transition [84], and mesenchymal precursor cells can further amplify the fibrotic process by triggering the Wnt-pathway [85]. Myofibroblasts are key elements in IPF and their differentiation can be also triggered by loss of telomerase activity [86]. Concurrently, Notch-signalling is crucial for myofibroblast differentiation and alveologenesis, and can also contribute to the differentiation of airway basal precursor cells [87].

But how can senescent pneumocytes abnormally trigger these pathways in IPF? Signals provided by the milieu of damaged alveolar cells can trigger a variety of reparative mechanisms, including the recruitment and stimulation of endogenous and exogenous progenitors [88], and it is possible to expect a severe derangement of this process in senescent alveoli. In several systems it has been demonstrated that cell senescence can trigger a "senescence-associated secretory phenotype", that is able to stimulate the production of proliferative and profibrotic mediators, including growth factors, cytokines, chemokines, and metalloproteinases [89], acting on neighbouring epithelial and mesenchymal cells thus perturbing their physiological crosstalk as previously proposed [16,17]. In line with this assumption, both Wnt- and Notch pathways have been shown to be activated by cell senescence, and epithelial mesenchymal transition and mobilization of beta-catenin are among the features characterising the senescence-related hyper-secretive phenotype [88-95]. In senescent alveoli mesenchymal and epithelial precursors could be the target of this deranged cascade of stimulatory signals, with eventual myofibroblast activation and bronchiolar remodelling. Senescent myofibroblasts in turn could be also stimulated to acquire a secretive phenotype, and this effect is likely to occur at short distance from damaged areas, thus contributing to produce the patchy distortion of pulmonary tissue characterising the "usual interstitial pneumonia" (UIP) pattern. Recently, we demonstrated that fibroblast foci are mainly located within micro-honeycombing lesions in IPF, at sites where basal cell airway precursors abnormally show over-expression of molecules involved in cell-motility (laminin-5 γ-2-chain and heath shock protein 27), and molecules that can be directly related to cellular senescence including p21^waf1 ^and p53 [96,97]. Small airways and alveolar epithelia are characterised by quite different renewal strategies at the molecular level, and telomere dysfunction and cellular senescence could be expected to act differently in these two compartments. Bronchiolar progenitors, located in the basal layer [98], express high levels of ΔN-p63+, a potent anti-apoptotic mediator that can interfere with the p53/p21 pathway and may potentially contrast cell senescence in basal cells [96,99]. Bronchiolar abnormal proliferation and honeycomb changes are common in IPF and can be considered as consequence of divergent behaviours in proximal and distal lung compartments [15,56,99]. In our view, exaggerated autocrine and paracrine activation of the Wnt- and Notch-pathways can in part explain honeycomb cyst formation, since proliferation and differentiation of basal cell precursors in small airways depend on the correct expression of these signalling pathways [98]. Further contribute to the aberrant bronchiolar proliferation and honeycomb cyst formation in IPF is likely provided by abnormalities affecting the production of airway mucins, as recently demonstrated [70,100]. Interestingly, disordered mucin production with increased MUC5B forms is also observed in the airway of COPD patients [101].

### Wnt and Notch signalling perturbation in COPD

Interestingly, the same signalling pathways involved in IPF (Wnt- and Notch), seem to have a relevant role also in COPD, but in the opposite way. In fact, both Wnt- and Notch- appear as significantly inhibited in COPD, rather than activated as observed in IPF [80-82,102,103], and this observation can explain why in emphysema enlarged alveoli are mainly covered by type-I differentiated pneumocytes and type II pneumocyte proliferation is minimal. In different systems in fact, the classical role of the Notch- and Wnt-signalling, acting in concert, is the maintenance of self-renewal potential of epithelial precursor cells and the regulation of cell differentiation [72-78], and the abnormal decrease of these pathways, as observed in emphysema, can be detrimental for the correct renewal of pulmonary parenchyma [102,103]. Accordingly, activation of the Wnt/beta-catenin pathway can attenuate experimental emphysema [104], and accelerated precursor cell senescence and dysfunction are related to aberrant Notch and Wnt-signalling, particularly affecting the correct differentiation of mesenchymal precursors [90-92].

The observed inhibition of Wnt-signalling in COPD may be ascribed to a variety of concurrent causes, including the smoking-related up-regulation of extracellular Wnt antagonists (e.g. secreted frizzled-related proteins) [103]. Interestingly, the Wnt-pathway is sensitive to mechanical stimuli in different systems, and it is considered to represent a key factor in mechano-transcription processes [105-108]. It is then possible to hypothesise that the decrease of elastic recoil occurring in emphysematous parenchyma may significantly contribute, in a vicious circle, to perpetuate the Wnt-signalling down-modulation. In IPF, on the other hand, Wnt activation could be amplified at sites where mechanical stress is higher (e.g. in the subpleural lower lung portions, typically affected in IPF), thus contributing to ongoing alveolar loss and fibrosis, as recently hypothesised [79].

All these data taken together, it is possible to hypothesise that in COPD the abnormal senescence of mesenchymal precursors can cause both an impaired production of extracellular matrix proteins (e.g. elastin), as well as a derangement of the interplay between signalling pathways that regulate alveologenesis (in particular Wnt and Notch). These abnormalities are likely sufficient to cause the progressive weakening of the scaffold interstitial structures sustaining the pulmonary parenchyma, with eventual alveolar dilatation and emphysema.

### Precursor cell senescence and inflammation in COPD-emphysema

Interestingly, the here proposed pathogenic scheme, centred on alveolar senescence and mesenchymal precursor cell insufficiency, can in part reconcile some controversial issues regarding the significance and role of inflammation and autoimmunity in the development of airway disease occurring in COPD [2,20,109-115]. Evidence has been in fact provided of a robust regulatory function of mesenchymal stem cells (MSC) on cells of both the innate and adaptive immune systems, and bone marrow derived MSC are able to inhibit the release of pro-inflammatory cytokines and also stimulate the functional activity of regulatory T-lymphocytes [116-119]. The regulatory functions of MSC is similar in different tissues [120,121], including the lung [Ricciardi M, et al: unpublished data]. It is then possible to hypothesise that in COPD the regulatory functions of senescent MSC can be variably impaired, with eventual triggering of "autoimmune-like" chronic inflammation in small airways, similar to that observed in the variety of lung diseases presenting as constrictive bronchiolitis in different settings (lung allografts, exposure to toxic substances, autoimmunity, post-viral, etc.) [122-126]. According to this hypothesis is the recent demonstration of a direct role of mesenchymal cell senescence and telomere dysfunction in causing airway inflammation in COPD [127]. In this scheme, the airway remodelling characterising COPD could be tentatively explained by the concurrence action of the inflammatory stimuli derived by the above described inhibition of MSC immune-modulatory role, and the proliferative response of airway epithelium to the chronic damaging effect of exogenous toxic substances (e.g. cigarette smoke).

## Conclusions

**Figure 2:**
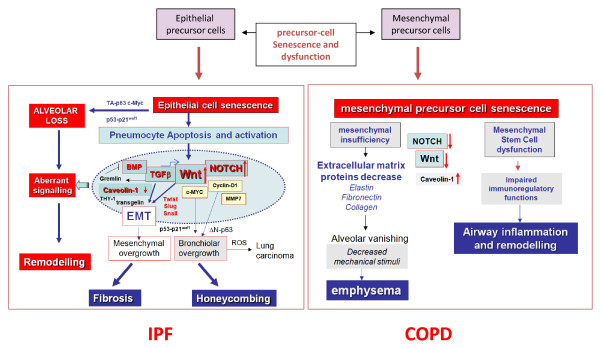
**Pathogenic scheme of IPF and COPD**. The complex effects of either epithelial (left), or mesenchymal (right) insufficiency on derangements of various signaling pathways in IPF and COPD as hypothesized in this review is summarized.

In summary, in both COPD and IPF a common pathogenic scheme can be traced where an accelerated cellular senescence determined by the "two hits" paradigm (genetic predisposition to cell senescence with the concurrence of tobacco smoke), determines an impaired regeneration of the lung parenchyma after damage. The divergence in two horns in this model is provided by the affected precursor cells (mesenchymal in COPD and emphysema, epithelial in IPF), the relevance of genetic background, as well as by the basic signalling pathways involved in the development of either emphysema or fibrosis (Wnt-, Notch-, etc.)(Figure 2). Both mechanisms could be involved in the cases with combined pulmonary fibrosis and emphysema [8,9].

The complexity of this network is difficult to be completely deciphered in both IPF and COPD, since beyond precursor cell senescence, as here described, other genetic predisposing factors and molecular mechanisms are likely involved in both diseases, including micro-RNA regulation [128-133]. Interestingly, perturbation of micro-RNAs can also affect TGF-beta, a potent profibrotic effector, that plays a relevant role in early lung development, has significant interconnections with the Wnt-pathway, and is involved in the pathogenesis of both IPF and COPD [129,133-136]. Another player in this complex scenario is likely represented by caveolin-1, the member of a protein family involved in the formation of cellular caveolae, that plays divergent roles in the development of IPF and emphysema, respectively [137-141].

These evolving concepts open new options to better understand the pathogenesis of both IPF and COPD, as far as the involvement of both parenchymal and small airway components are concerned [142,143], and also new perspectives for alternative treatment options, including drugs specifically addressing some of the mechanisms described in this review. The high relevance of the type of cell precursor involved in the two diseases is emphasised, since future efforts should be focused on their pharmacological protection or specific replacement [144-149].

## Competing interests

The authors declare that they have no competing interests.

## Authors' contributions

All Authors participated in the design of the review and helped to draft the manuscript. All authors read and approved the final manuscript.
